# Digital Spatial Profiling Reveals Functional Shift of Enterochromaffin Cell in Patients With Ulcerative Colitis

**DOI:** 10.3389/fcell.2022.841090

**Published:** 2022-04-08

**Authors:** Dongping Lyu, Guanjun Kou, Shiyang Li, Lixiang Li, Bing Li, Ruchen Zhou, Xiaoxiao Yang, Wenyu Tian, Yanqing Li, Xiuli Zuo

**Affiliations:** ^1^ Department of Gastroenterology, Qilu Hospital, Shandong University, Jinan, China; ^2^ Laboratory of Translational Gastroenterology, Qilu Hospital, Shandong University, Jinan, China; ^3^ Advanced Medical Research Institute, Shandong University, Jinan, China; ^4^ Key Laboratory for Experimental Teratology of Ministry of Education, Shandong University, Jinan, China; ^5^ Robot Engineering Laboratory for Precise Diagnosis and Therapy of GI Tumor, Qilu Hospital, Shandong University, Jinan, China

**Keywords:** enterochromaffin cell, ulcerative colitis, digital spatial profiling, chromogranin B, single-cell RNA-seq

## Abstract

As a major component of the enteroendocrine system, enterochromaffin (EC) cells play a key role in ulcerative colitis (UC). However, the scarcity of EC cells has limited the investigation of their function. In this study, we applied digital spatial profiling to acquire transcriptomic data for EC cells and other epithelial cells from colonoscopic biopsy samples from eight patients with UC and seven healthy controls. Differential expression analysis, gene set enrichment analysis, and weighted gene coexpression network analysis were performed to identify differentially expressed genes and pathways and coexpression networks. Results were validated using an online dataset obtained by single-cell RNA sequencing, along with immunofluorescence staining and quantitative real-time PCR. In healthy participants, 10 genes were significantly enriched in EC cells, functionally concentrated in protein and bioamine synthesis. A coexpression network containing 17 hub genes, including *TPH1*, *CHGA*, and *GCLC*, was identified in EC cells. In patients with UC, EC cells gained increased capacity for protein synthesis, along with novel immunological functions such as antigen processing and presentation, whereas chemical sensation was downregulated. The specific expression of *CHGB* and *RGS2* in EC cells was confirmed by immunofluorescence staining. Our results illuminate the transcriptional signatures of EC cells in the human colon. EC cells’ newly observed functional shift from sensation to secretion and immunity indicates their pivotal role in UC.

## Introduction

Ulcerative colitis (UC) is a major form of inflammatory bowel disease with a clinical manifestation of bloody stool, abdominal pain, and diarrhea (Ordás et al., 2012). The burden of UC is increasing worldwide, especially in developing countries (Ng et al., 2017). Many factors have been reported to be involved in UC, including genetic susceptibility, immune system dysregulation, and diet (Ordás et al., 2012; Kobayashi et al., 2020). However, understanding of the pathology of UC remains inadequate.

Enterochromaffin (EC) cells are a major component of the enteroendocrine system. Although they only constitute about 1% of the intestinal epithelium (Latorre et al., 2016), EC cells play a crucial role in gut homeostasis. They are the main producers of 5-hydroxytryptamine (5-HT, also known as serotonin) in the human body and also secrete various other enteroendocrine hormones, including glucose-dependent insulinotropic polypeptide (GIP), glucagon-like peptide 1 (GLP-1), and secretin (Reynaud et al., 2016; Martins et al., 2017; Fazio Coles et al., 2020). Another role of EC cells is to receive stimuli from the gastrointestinal tract. A variety of chemical receptors have been identified in EC cells, including the fatty acid receptors Ffar2, Ffar3, and Olfr558 (Akiba et al., 2015; Martin et al., 2017; Lund et al., 2018), as well as the bile acid receptor TGR5 (Kidd et al., 2008). Recent studies have also shown that EC cells can sense mechanical signals *via* Piezo2 (Wang F. et al., 2017; Alcaino et al., 2018) and ADORA2B (Chin et al., 2012; Dammen et al., 2013). In addition, Bellono et al. (2017) reported direct contact between EC cells and neural synapses *via* neuropods in mice, further indicating EC cells’ role as relays for environmental and intrinsic signals. Moreover, there is growing evidence of a deep relationship between EC cells and the immune system, mediated *via* 5-HT and chromogranin A (Wang Y. et al., 2017; Eissa et al., 2018; Ito et al., 2019; Eissa et al., 2020). Previous studies have also reported that UC is associated with a rise in the number of EC cells (El-Salhy et al., 1997); however, the exact role of EC cells in UC is still unknown.

The scarcity and scattered distribution of EC cells has severely limited the investigation of their function using currently available methods. Previous studies of EC cells in patients with UC have mainly focused on enhancement of the secretory function, particularly with regard to 5-HT and chromogranin A (Strid et al., 2013), disregarding other aspects of EC cell function. In recent years, the rapid progression in technologies such as fluorescence-activated cell sorting and single-cell RNA sequencing (RNA-seq) has enabled transcriptomic analyses of EC cells in human intestinal organoids and in mice (Gehart et al., 2019; Piccand et al., 2019; Beumer et al., 2020); however, knowledge of the biology of human EC cells *in situ* remains inadequate. Although several recent studies have involved single-cell RNA-seq of colonoscopic biopsy specimens from patients with UC (Parikh et al., 2019; Smillie et al., 2019; Li et al., 2021), these studies have produced few findings regarding EC cells, probably due to limited sequencing depth and EC cell scarcity.

In this study, we investigated molecular expression patterns in EC cells by performing digital spatial profiling (DSP) of colonoscopic biopsy specimens from both patients with UC and healthy controls, revealing a distinct transcriptomic signature of EC cells, and that EC cells in patients with UC may display a functional shift from chemical sensation to secretion and antigen presentation.

## Materials and Methods

### Participants

A total of 42 patients diagnosed with active UC and 36 healthy control participants with matched age and sex were recruited at Qilu Hospital of Shandong University. The diagnosis of UC was based on well-established clinical, endoscopic, and histopathological criteria. Biopsy specimens of patients with UC were collected from the mucosa with mild to moderate inflammation. All control participants were individuals who underwent colonoscopy for screening purposes, with a macroscopically normal colon and no clinical symptoms. The experimental procedures were approved by the ethical committee of Qilu Hospital of Shandong University. All participants gave written informed consent before biopsy.

### Sample Preparation and Tissue Microarray Construction

As the DSP sequencing required a sufficient amount of RNA for detection by probes, the distribution of EC cells in each specimen was screened in advance using immunohistochemical methods as follows. Biopsy specimens were collected from the inflamed mucosa of the sigmoid colon or rectum and immediately fixed in 4% neutral buffered polyformaldehyde overnight. After dehydration and embedding, 4-μm sections of each specimen were examined to determine the number of EC cells. Sections were deparaffinized, boiled in sodium citrate antigen retrieval solution for 15 min, and stained with the anti–5-HT antibody (Supplementary Table S1) and hematoxylin. Specimens with fewer than 30 EC cells in any area of 660 × 785 μm were excluded from further procedures, leaving 55 suitable specimens, which were assembled into tissue microarray (TMA) blocks.

### *In situ* Hybridization and Digital Spatial Profiling

To prepare samples for DSP, 4-μm sections from the TMA blocks were deparaffinized and then boiled in Tris-EDTA buffer in a pressure cooker (Bio SB, Santa Barbara, CA, USA) to expose RNA targets. The sections were digested to remove RNA-binding proteins (GeoMx DSP RNA Slide Prep Kit for FFPE; NanoString, Seattle, WA, USA), fixed in 16% formaldehyde at room temperature for 5 min, and incubated overnight in an autostainer with probes for 18,676 endogenous targets (GeoMx Panel and Seq Code kit, NanoString). The probes were connected with DNA oligos [consisting of an RNA analyte identifier, a UMI (unique molecular identifier) barcode, and primer binding sites] by a UV-photocleavable linker to ensure a fine spatial profiling of the samples.

Prepared slides were further incubated with anti-EPCAM for epithelial cells or anti–5-HT for EC cells (Supplementary Table S1), as well as Syto 13 (NanoString) for nuclear identification. After incubation at room temperature for 1 h, slides were washed and stained with fluorescent secondary antibodies at 37°C for 30 min. Finally, slides were loaded into a GeoMX Digital Spatial Profiler (NanoString) and scanned.

After scanning, regions of interest (ROIs) were selected on the basis of immunofluorescence signal, with each ROI covering either an EPCAM+ 5-HT+ area or an EPCAM+ 5-HT− area. ROIs were divided into four groups depending on whether they contained EC cells from patients with UC (EC-UC), EC cells from controls (EC-Con), background epithelial cells from patients with UC (Epi-UC), or background epithelial cells from controls (Epi-Con). A total of 19 ROIs, each measuring 660 × 785 μm, were selected: eight for EC-UC, seven for EC-Con, two for Epi-UC, and two for Epi-Con (Supplementary Figure S1).

After ROI selection, DNA oligos in the ROIs were photocleaved using the GeoMX Digital Spatial Profiler and aspirated. To prepare the aspirates for PCR, Illumina adapter sequences and unique dual sample indices were added. After pooling, purification, and quality control (QC), the library was prepared and sequenced with an Illumina NovaSeq 6,000 next-generation sequencing (NGS) platform (Illumina, San Diego, CA, USA), using a dual-index workflow (Decalf et al., 2019; Merritt et al., 2020; Wang et al., 2021).

### Pre-Processing of the Transcriptomic Data

The raw reads were subjected to an NGS analysis pipeline as follows. First, sequencing QC was performed. Reads were removed if more than 50% of bases had a Phred quality score ≤ 19. If the adapter length was greater than 5 bp, then both paired-end reads were removed. Next, the adapters were removed, and the paired-end reads were merged. The reads were aligned to the tag sequence identifier barcodes from the DSP readout, and the PCR duplicates were removed by UMI matching. QC failure was defined as an average raw count per target below 6. The data were scaled and normalized on the basis of the top 25% most expressed genes, which were used to generate heatmaps of overall gene expression. The limit of quantification (LOQ) of each ROI was calculated on the basis of the formula below. GeoMean, geometric mean; NegProbe, negative probe; GeoSD, geometric standard deviation.

$$\text{LOQ }=\text{ GeoMean}\left(\text{NegProbe}\right)\times \text{GeoSD}{\left(\text{NegProbe}\right)}^{2}$$

For dimensionality reduction, principal component analysis (PCA) was performed using the prcomp function in R. Multivariate analysis of the PCA results was performed using the R package vegan 2.5–7 (https://cran.r-project.org/web/packages/vegan/). The package ggalt 0.4.0 (https://cran.r-project.org/web/packages/ggalt/) was used to generate circles. All data generated using R were visualized using ggplot2 3.3.5 (Wickham, 2016), unless otherwise mentioned.

All bioinformatic analyses were performed using R 4.0.5 and RStudio 1.4.1106.

### Differential Expression Analysis of Biopsy Specimen Data

Analysis of differential expression between ROI groups was performed using the R package DESeq2 1.32.0 (Love et al., 2014). *p*-values were adjusted using the Benjamini–Hochberg method, with the significance cutoff set to 0.05. The cutoff for log_2_(FoldChange) was 1. The results were visualized using the R packages pheatmap 1.0.12 (https://cran.r-project.org/web/packages/pheatmap/) and UpSetR 1.4.0 (Conway et al., 2017).

### Gene Set Enrichment Analysis

To explore differences in pathway enrichment level, gene set enrichment analysis (GSEA) was conducted using the R package clusterProfiler 4.0.5 (Yu et al., 2012). Kyoto Encyclopedia of Genes and Genomes (KEGG) pathway categories were examined, as well as all three subontologies (molecular function, biological process, and cellular component) of the Gene Ontology (GO) hierarchy; *p*-value significance cutoff was set to 0.05. The “simplify” function of clusterProfiler was used to remove redundant items.

### Weighted Gene Coexpression Network Analysis

Weighted gene coexpression network analysis (WGCNA) was performed using the R package WGCNA 1.70–3 (Langfelder and Horvath, 2008) to identify gene coexpression networks and hub genes. First, we used the normalized data matrix to calculate the soft threshold. Scale-free network construction and module detection were then performed with the optimal power value automatically selected. A cluster dendrogram was plotted to visualize the detected gene coexpression modules. Associations between gene modules and sample parameters (cell type, UC status, and ROI group) were calculated and filtered. Heatmaps of relationships between modules and sample parameters were generated for modules with at least one *p*-value ≤ 0.05.

One module, designated Green3, was found to be significantly associated with EC cells. The module membership and gene significance of each gene in Green3 were determined and plotted against each other. These genes were then extracted for overrepresentation analysis (ORA) based on GO terms. Rich factors were calculated as defined in a previous study (Wu et al., 2021). Network data were exported to Cytoscape 3.8.2 (Shannon et al., 2003). Cytohubba version 0.1 (Chin et al., 2014) was used to identify the hub genes of Green3.

### Transcription Factor Enrichment Analysis

Transcription factor enrichment analysis was performed on ChIP-X Enrichment Analysis 3 (ChEA3) platform (Keenan et al., 2019). The prediction of active transcriptive factors (TFs) was predicted on the basis of the differentially expressed genes (DEGs) between EC-UC and EC-Con. The results were ranked according to the mean rank method.

### Differential Expression Analysis of Single-Cell Transcriptomic Data

Single-cell transcriptomic data for colonic cells from patients with UC and healthy individuals were downloaded from Single Cell Portal (https://singlecell.broadinstitute.org/), accession number SCP259. Seurat 4.0.5 (Hao et al., 2021) was used for data processing and clustering. Cells were considered low-quality and filtered out if the estimated number of genes was between 200 and 7,000, the proportion of reads mapping to the mitochondria was below 75%, the proportion of reads mapping to the ribosome was below 50%, or total RNA molecule count was below 40,000. Only genes detected in at least five cells were considered for subsequent analyses.

For cell clustering, we followed the standard procedure for Seurat. First, we normalized the data and identified the top 2,000 variable features and then scaled the data based on these features. PCA-based dimensionality reduction was then performed with dimensions set to 30, followed by the integration of samples from different individuals using Harmony 0.1.0 (Korsunsky et al., 2019). The results of the integration were used to find unsupervised cell clusters, with dimensions set to 30 and resolution set to 0.3. Cell clusters were visualized using Uniform Manifold Approximation and Projection.

We distinguished enteroendocrine cells based on their increased expression of *CHGA*, *TPH1*, and *PCSK1N*. To identify EC cells, we isolated the data for enteroendocrine cells and subjected them to the procedure of normalization, integration, and clustering described above. Cells were identified as EC cells if they were high in *TPH1*, low in *PYY*, and low in *GCG*.

Because of the low number of EC cells, only genes found in more than 10% of cells were considered for differential expression analyses. Conserved markers of EC cells were identified using the FindConservedMarkers function, whereas the FindMarkers function was used to compare healthy control, inflamed UC, and non-inflamed UC samples and identify DEGs. Genes were considered significantly differentially expressed at log_2_(FoldChange) ≥ 1 and adjusted *p*-value ≤ 0.05.

### Immunofluorescence Staining

Slides were deparaffinized and subjected to antigen retrieval as previously described and then incubated with primary antibodies at 4°C overnight. After three washes with phosphate-buffered saline, slides were incubated with secondary antibodies for 1 h at room temperature, mounted with antifade mountant, and observed under a fluorescence microscope. Antibodies used in this experiment are shown in Supplementary Table S1.

### Quantitative Real-Time Polymerase Chain Reaction

Total RNA was isolated from colonoscopy biopsy specimens using TRIzol (Invitrogen, CA, USA) according to the manufacturer`s instructions. Reverse transcription was conducted using the Reverse Transcription Kit (Takara, Osaka, Japan). Quantitative real-time PCR (qRT-PCR) was then performed using the ReverTra Ace qPCR RT Kit with SYBR Green (Toyobo, Osaka, Japan) in triplicates on an Applied Biosystems StepOne Real-Time PCR System (Thermo Fisher, MA, USA). With *GAPDH* as the internal control target, the expression levels were calculated on the basis of the 2^−ΔΔCt^ method. The primer sequences for CHGB amplification are (forward primer) 5′-CGA​GGG​GAA​GAT​AGC​AGT​GAA-3′ and (reverse primer) 5′-CAG​CAT​GTG​TTT​CCG​ATC​TGG-3′.

## Results

### Transcriptomic Profiling of Enterochromaffin Cells by Digital Spatial Profiling

**FIGURE 1:**
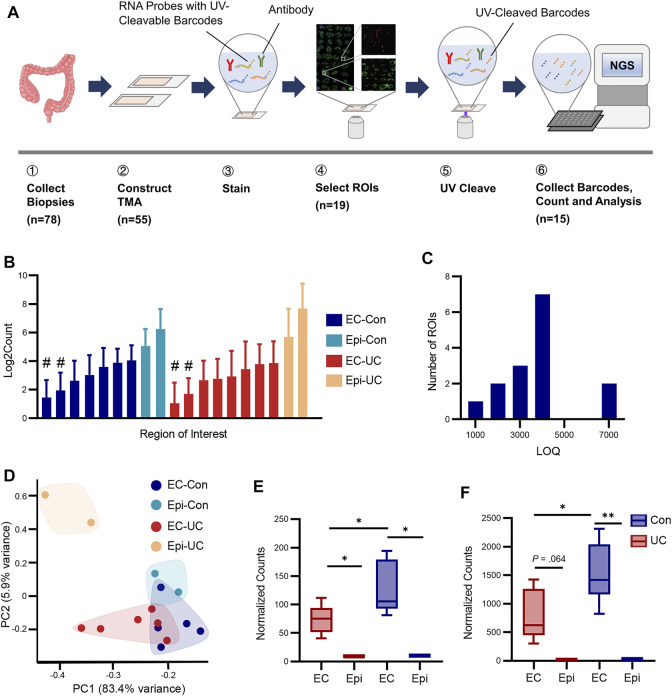
Digital spatial profiling of EC cells from human colon biopsy. **(A)** Schematic workflow of digital spatial profiling. Biopsy specimens were collected and assembled into TMAs. The sections were incubated with immunofluorescence antibodies (for cell type identification) and RNA probes with UC-cleavable barcodes. After ROI selection, barcodes within each ROI were cleaved from probes by a fine beam of UV ray and collected for NGS sequencing. **(B)** Average raw count of targets in each ROI. The *y*-axis was log2-transformed. EC-Con, EC cells from healthy participants; Epi-Con, epithelium (excluding EC) from healthy participants; EC-UC, EC cells from patients with ulcerative colitis; Epi-UC, epithelium from patients with UC. # indicates QC failure. **(C)** To determine how many genes were detected in each ROI, the number of targets above the limit of quantification (LOQ) in each ROI was calculated. **(D)** Principal component analysis (PCA) of ROIs from each group. **(E,F)** Expression levels of *TPH1* and *CHGA*. Differences between groups were analyzed using Student’s *t*-test (two-tailed). *p < 0.05 and **p < 0.01.

To characterize the gene expression profile of EC cells in the human large intestine, we collected 78 colonoscopic biopsy specimens. To ensure a sufficient amount of RNA for detection by DSP probes, the distribution of EC cells in each sample was screened in advance using immunohistochemical methods. Two TMAs containing 55 suitable biopsy specimens were subjected to standard DSP procedures. Areas with a relatively abundant distribution of EC cells were selected as ROIs for subsequent sequencing (Figure 1A, Supplementary Figure S1).

After QC, 15 ROIs were selected for further analysis (Table 1). To evaluate our sequencing efficiency, we calculated the average raw target count and LOQ for each sample (Figures 1B,C). The median number of genes above LOQ was 3,627, ranging from 1,355 to 7,267. PCA revealed distinctive transcriptomic patterns among ROI groups (Figure 1D). Permutational analysis of variance showed significant differences between patients with UC and healthy controls (Pr = 0.008, *R* ^2^ = 0.138), but no significant differences between cell types (Pr = 0.447, *R* ^2^ = 0.067), possibly due to the small number of background epithelium samples. Biopsy location, a covariate parameter, showed no significance (Pr = 0.994, *R* ^2^ = 0.031).

**TABLE 1:** Demographic and clinical characteristics of the participants in each group.

	Con-EC (*n* = 5)	Con-Epi (*n* = 2)	UC-EC (*n* = 6)	UC-Epi (*n* = 2)
Sex, *n* (%)				
Male	**3 (60.0)**	**0 (0.0)**	**2 (33.3)**	**0 (0.0)**
Female	**2 (40.0)**	**2 (100.0)**	**4 (66.7)**	**2 (100.0)**
Age, years, mean ± SD	**40.2 ± 14.1**	**47.0 ± 18.4**	**44.2 ± 22.1**	**46.5 ± 9.2**
BMI, mean ± SD	**24.5 ± 3.0**	**23.0 ± 2.4**	**20.3 ± 1.2**	**20.1 ± 3.0**
Location of biopsy, *n* (%)				
Sigmoid Colon	**0 (0.0)**	**1 (50.0)**	**1 (16.7)**	**1 (50.0)**
Rectum	**5 (100.0)**	**1 (50.0)**	**5 (83.3)**	**1 (50.0)**
Disease duration, months, mean ± SD	N.A.	N.A.	**20.8 ± 32.2**	**18.0 ± 11.3**
Disease extent, *n* (%)				
E1 (proctitis)			**2 (33.3)**	**0 (0.0)**
E2 (left sided)	N.A.	N.A.	**2 (33.3)**	**1 (50.0)**
E3 (extensive)			**2 (33.3)**	**1 (50.0)**
Concomitant medication, *n* (%)				
5-ASA			**4 (66.7)**	**2 (100.0)**
Corticoids	N.A.	N.A.	**0 (0.0)**	**1 (50.0)**
None			**2 (33.3)**	**0 (0.0)**
Mayo endoscopic score, mean ± SD	N.A.	N.A.	**2.2 ± 0.8**	**2.5 ± 0.7**
Mayo score, mean ± SD	N.A.	N.A.	**6.8 ± 1.7**	**9.5 ± 0.7**

The data was displayed either as mean ± SD, or number (percentage). BMI, body mass index. 5-ASA, 5-aminosalicylic acid.

To validate the sequencing results, we investigated the expression levels of *TPH1* and *CHGA*, two major markers of EC cells. As expected, the expression of *TPH1* and *CHGA* was significantly higher in both EC-UC and EC-Con groups than in background epithelium (Figures 1E,F). The level of *TPH1* and *CHGA* in EC cells from patients with UC was also higher than in EC cells from controls, which was consistent with previous studies (El-Salhy et al., 1997; Sciola et al., 2009).

### Adaptation of Enterochromaffin Cells for Protein Synthesis and Secretion

**FIGURE 2:**
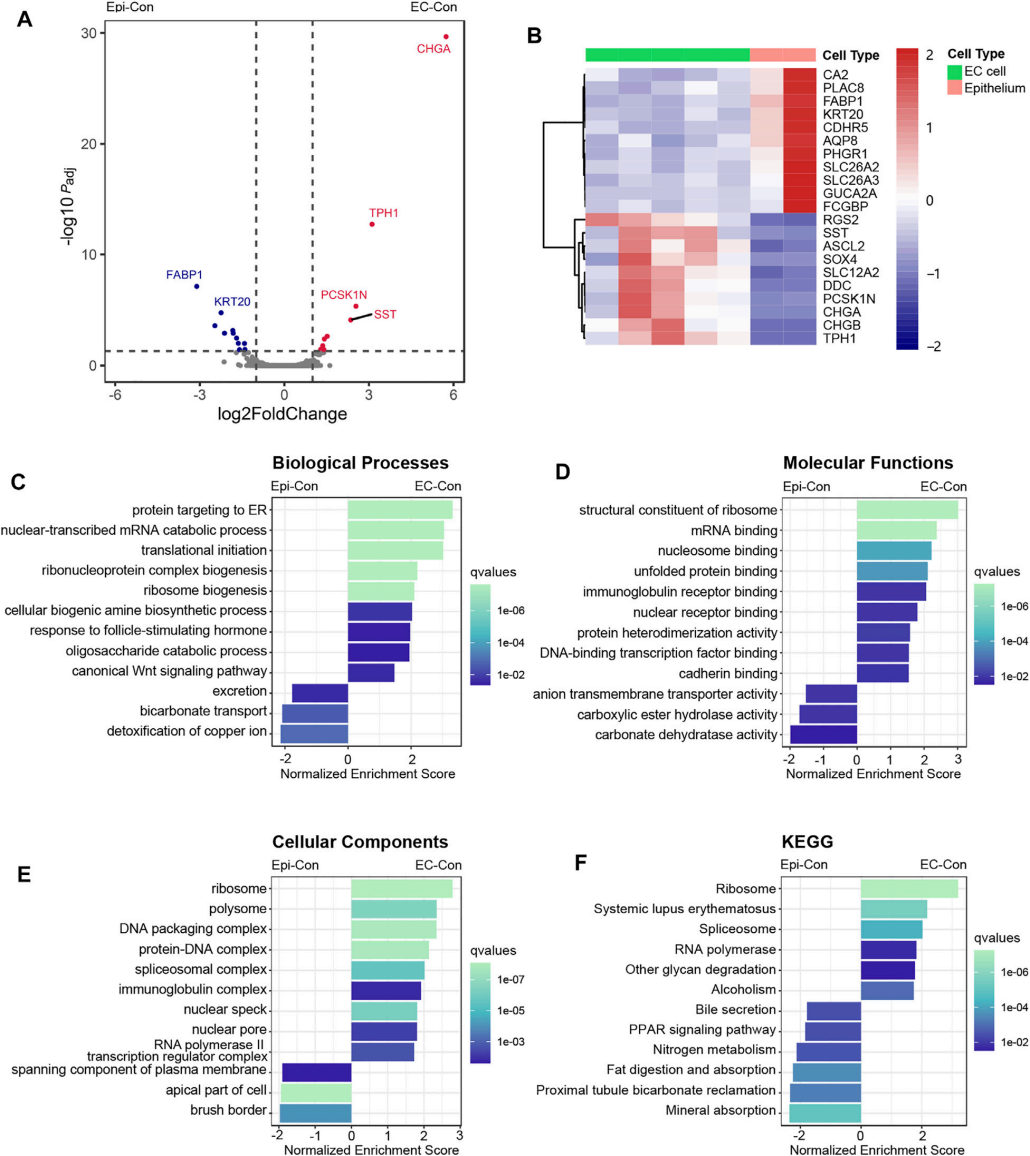
Comparison between EC cells and background epithelium in healthy participants, revealing the distinctive expression patterns of EC cells. Participants, revealing the distinctive expression patterns of EC cells. **(A)** Volcano plot of the analysis of differential expression between the EC-Con and Epi-Con groups. Vertical dotted lines represent log2FoldChange values of 1 and −1. The horizontal dotted line represents adjusted *P* = 0.05. *P*_adj_, adjusted *p*-value. **(B)** Heatmap of DEGs between EC cells and background epithelium in healthy participants. **(C–F)** Results of GSEA comparing the EC-Con and Epi-Con groups. Analysis was performed using the Gene Ontology categories of biological process **(C)**, molecular function **(D)**, and cellular component **(E)**, as well as KEGG pathways **(F)**. The length of each bar indicates the normalized enrichment score, and the color indicates q values of each term.

To investigate the function of EC cells in healthy tissue, we compared the EC-Con and Epi-Con groups using differential expression analysis. A total of 10 genes were significantly enriched in EC cells compared to background epithelium, whereas 11 genes were enriched in background epithelium (Figures 2A,B, Supplementary Table S2). The most enriched gene in EC cells was *CHGA*, encoding chromogranin A, a precursor of several secretory peptides. *SST* and *CHGB*, two other genes encoding secretory peptides, were also enriched. As expected, EC cells were also enriched in two members of the tryptophan pathway (*TPH1* and *DDC*), which are essential for 5-HT synthesis. The transcription factors *SOX4* and *ASCL2* were also enriched in EC cells. Genes enriched in background epithelium included *FABP1*, *SLC26A3*, *KRT20*, and *GUCA2A*, among others.

We then used GSEA to investigate the function of EC cells at the pathway level. In the GO biological process category, there was remarkable enrichment of genes annotated with the terms “protein targeting to ER,” “nuclear-transcribed mRNA catabolic process,” and “translation initiation,” accounting for major processes in gene expression and protein synthesis (Figure 2C). In the GO molecular function category, enriched genes were annotated with the terms “structural constituent of ribosome,” “mRNA binding,” “nucleosome binding,” and “unfolded protein binding” (Figure 2D), whereas in the GO cellular component category, there was enrichment of genes annotated with the terms “ribosome,” “polysome,” and “DNA packaging complex” (Figure 2E). KEGG pathway analysis also found enrichment in genes associated with the ribosome pathway (Figure 2F). As for the background epithelium, the most enriched pathways were those related to absorption and structures for absorption; absorption is the main role of enterocytes, which account for the vast majority of intestinal epithelial cells.

### Identification of Hub Genes in the Coexpression Networks of Enterochromaffin Cells

**FIGURE 3:**
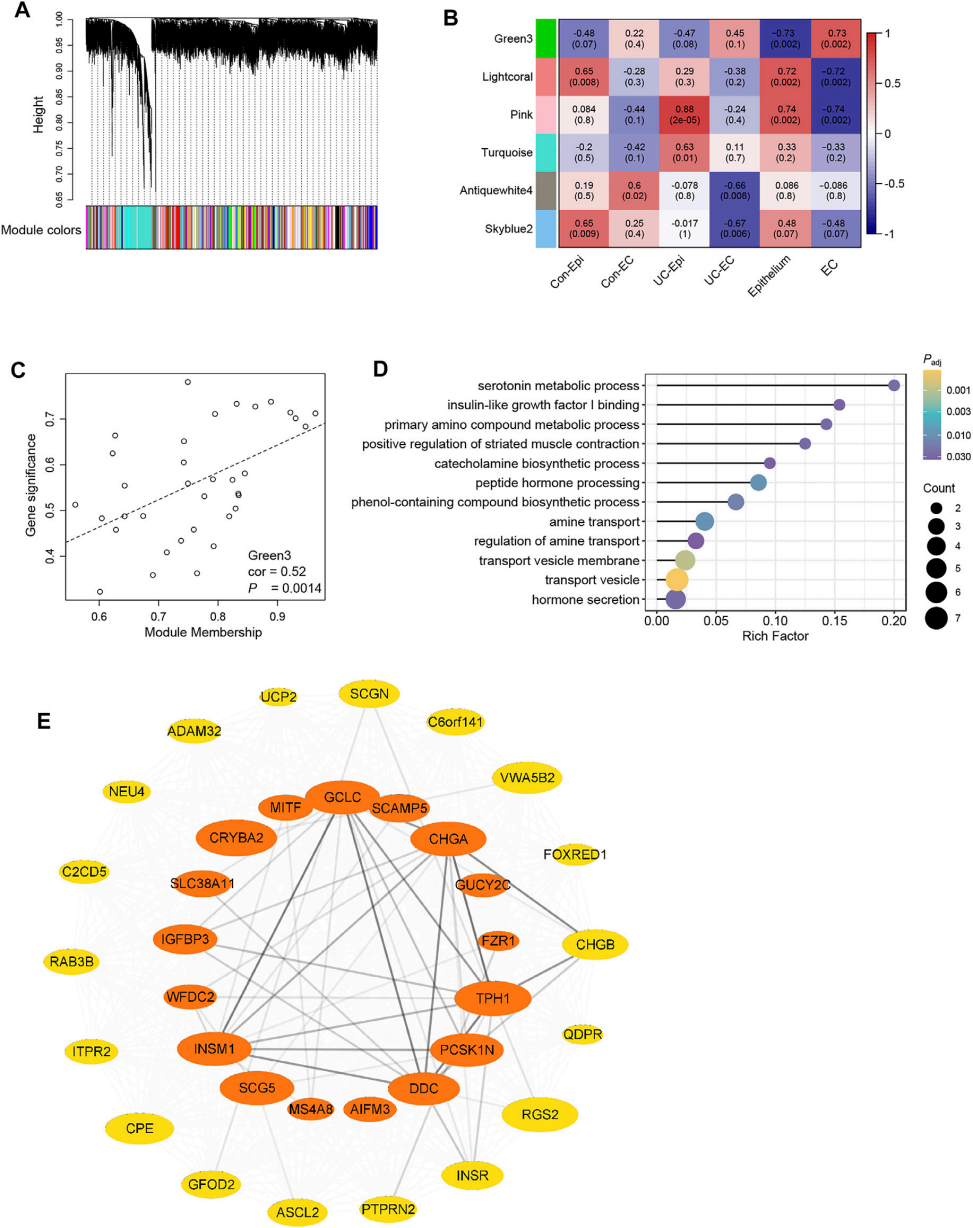
Identification of coexpression networks and hub genes of EC cells using WGCNA. **(A)** Cluster dendrogram of 149 coexpression modules detected by WGCNA. The color row underneath the dendrogram shows the module assignment determined by the dynamic tree cut **(B)** Associations between coexpression modules and sample parameters (group and cell type). Module names are represented by colors. The upper number in each block is the correlation coefficient, and the lower number in parentheses is the *p*-value. Only modules with at least one significant association (*p*-value < 0.05) were shown. **(C)** Correlations between module membership and gene significance weight for genes in the Green3 module, indicating that hub genes of the Green3 module also tend to be highly correlated with weight. cor, correlation; *P*, *p*-value. **(D)** Overrepresentation analysis (ORA) of genes in the Green3 module, analyzed using GO terms. Size of the rounds indicates gene count for each term, whereas the color indicates the *P*_adj_ (adjusted *p*-value) of each term. **(E)** Coexpression network of the genes in Green3. Orange indicates hub genes for EC cells; the other genes are plotted in yellow. Ellipse size represents the weight of the gene in the network, and the transparency of lines between genes represents the strength of gene interaction.

To further elucidate the transcriptomic patterns associated with EC cells, we used WGCNA to identify gene coexpression networks in these cells. A total of 149 coexpression modules were detected by automatic one-step network construction (Figure 3A). Associations between modules and sample parameters (ROI group and cell type, in particular) were calculated (Figure 3B). The module designated Green3, containing 35 genes, was found to be strongly associated with EC cells (correlation coefficient = 0.73, *P* = 0.002). In addition, genes in this module were strongly associated with the module as a whole (Figure 3C), further suggesting the important role of Green3 in EC cells.

We then focused on the function of the genes in Green3. ORA was conducted using GO terms (Figure 3D). The results showed significant enrichment of genes annotated with the terms “serotonin metabolic process,” “insulin-like factor I binding,” and “primary amino compound metabolic process.” Pathways involved in peptide hormone synthesis and secretion were also enriched, consistent with the neuroendocrine role of EC cells.

Next, we used Cytoscape and Cytohubba to identify 17 hub genes in the Green3 module, including the common enteroendocrine markers *TPH1*, *CHGA*, *PCSK1N*, and *SCG5* (Figure 3E). Although absent from our DEG list, other endocrine-associated genes such as *SCAMP5* and *IGFBP3* also appeared among the hub genes. Unexpectedly, genes rarely considered in studies of the enteroendocrine system, such as *CRYBA*, *SLC38A11*, and *MIFT*, were also identified as hub genes.

### Distinctive Expression Patterns of Enterochromaffin Cells in Ulcerative Colitis

**FIGURE 4:**
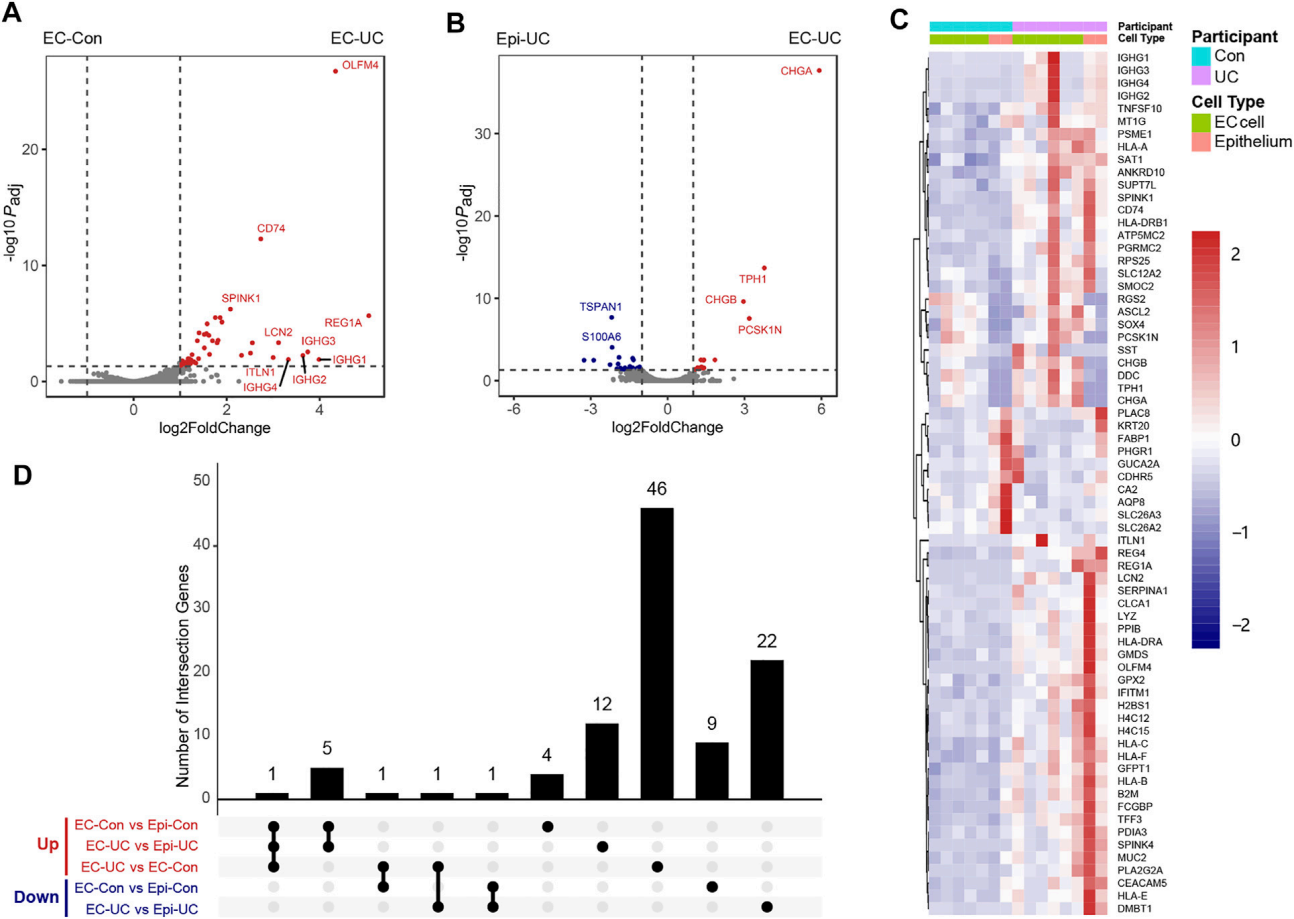
DEG analysis of EC cells in patients with ulcerative colitis. **(A)** Comparison of the transcriptomes of EC cells between patients with UC and controls. Vertical dotted lines represent log2FoldChange values of 1 and −1. The horizontal dotted line represents adjusted *P* = 0.05. *P*_adj_, adjusted *p*-value. **(B)** Comparison of the transcriptomes of EC cells and background epithelium in patients with UC. **(C)** Overall heatmap of genes identified in DEG analyses (EC-Con vs. Epi-Con, EC-UC vs. Epi-UC, and EC-UC vs. EC-Con). **(D)** Overlap of DEGs identified in the three analyses mentioned above. The upregulation (Up, in red) and downregulation (Down, in blue) of DEGs was shown, respectively. No genes were downregulated in EC-UC compared to EC-Con.

In this study, we sought to determine how EC cells behave under the conditions of UC. Differential expression analysis revealed that, compared to the EC-Con group, the EC-UC group showed significant upregulation of 49 genes and downregulation of none (Figure 4A, Supplementary Table S2). Most of the upregulated genes, such as *REG1A*, *OLFM4*, and *CD74*, were associated with inflammation and were also upregulated in the Epi-UC group (Figures 4C,D).

We also compared DEGs between EC-UC and Epi-UC groups (Figure 4B, Supplementary Table S2), finding that 18 genes were enriched in the EC-UC group. These included six genes that were also enriched in EC cells from healthy controls, as well as several that were not, including genes encoding transcription factors (*NEUROD1*, *INSM1*, and *MLXIPL*) and secretion-related genes (*SCG5* and *CPE*).

As expected, there was significant overlap among the three sets of DEGs found by the EC-Con/EC-UC, EC-UC/Epi-UC, and EC-CON/Epi-Con comparisons. Therefore, we generated a heatmap containing all three sets of DEGs to enable an intuitive understanding of their relationships (Figure 4C), together with an UpSet plot to describe the overlap among the three sets of DEGs in detail (Figure 4D). Six genes (*TPH1*, *CHGA*, *CHGB*, *RGS2*, *DDC*, and *PCSK1N*) were enriched in EC cells under both UC and healthy conditions, with *CHGB* being significantly upregulated in UC.

To explore the possible TFs in EC cell that regulate the genes upregulated in UC, we used ChEA3 for TF enrichment analysis. The top 10 TFs were shown in Table 2. Notably, *ISX* and *ATOH1* were related to the upregulation of *CHGB* in EC cells.

**TABLE 2:** The results of transcriptive factor enrichment analysis from ChEA3, compared between EC-UC and EC-Con.

Rank	TF	Score	Overlapping genes
1	MYRFL	7	SPINK4, MUC2, GMDS, DMBT1, REG4, PLA2G2A, TFF3, CLCA1, OLFM4
2	IRF9	7.5	CD74, IFITM1, HLA-B, HLA-C, HLA-A, HLA-F, SUPT7L, HLA-E, PSME1, TNFSF10, LCN2, PPIB, B2M
3	ISX	9.333	SPINK4, FCGBP, GPX2, SPINK1, REG4, PLA2G2A, ITLN1, OLFM4, MUC2, DMBT1, GMDS, CEACAM5, TFF3, LCN2, CLCA1, CHGB
4	IRF7	13.67	CD74, IFITM1, HLA-B, PSME1, TNFSF10, LCN2, HLA-C, HLA-A, HLA-F, B2M, HLA-E
5	BATF2	17.67	CD74, IFITM1, HLA-B, PSME1, TNFSF10, LCN2, HLA-C, HLA-A, HLA-F, B2M, HLA-E
6	CDX1	17.67	SPINK4, FCGBP, CD74, GPX2, SPINK1, REG4, PLA2G2A, ITLN1, OLFM4, MUC2, GMDS, DMBT1, CEACAM5, TFF3, CLCA1
7	IRF1	23	PDIA3, CD74, IFITM1, SPINK1, HLA-B, HLA-C, HLA-A, HLA-F, SAT1, HLA-E, RPS25, TNFSF10, PSME1, ANKRD10, B2M
8	SP100	23.67	CD74, IFITM1, PSME1, TNFSF10, HLA-DRA, HLA-A, HLA-F, B2M, HLA-DRB1, HLA-E
9	ATOH1	24.33	SPINK4, FCGBP, SMOC2, MUC2, REG4, CEACAM5, PLA2G2A, TFF3, CLCA1, OLFM4, CHGB
10	ARID5A	25.5	CD74, IFITM1, HLA-B, HLA-C, HLA-A, HLA-F, B2M, HLA-DRB1, HLA-E

The results were ranked according to the mean rank method. TF, transcriptive factor.

### Shift in Enterochromaffin Cell Function in Ulcerative Colitis From Chemical Sensation to Secretion and Antigen Presentation

**FIGURE 5:**
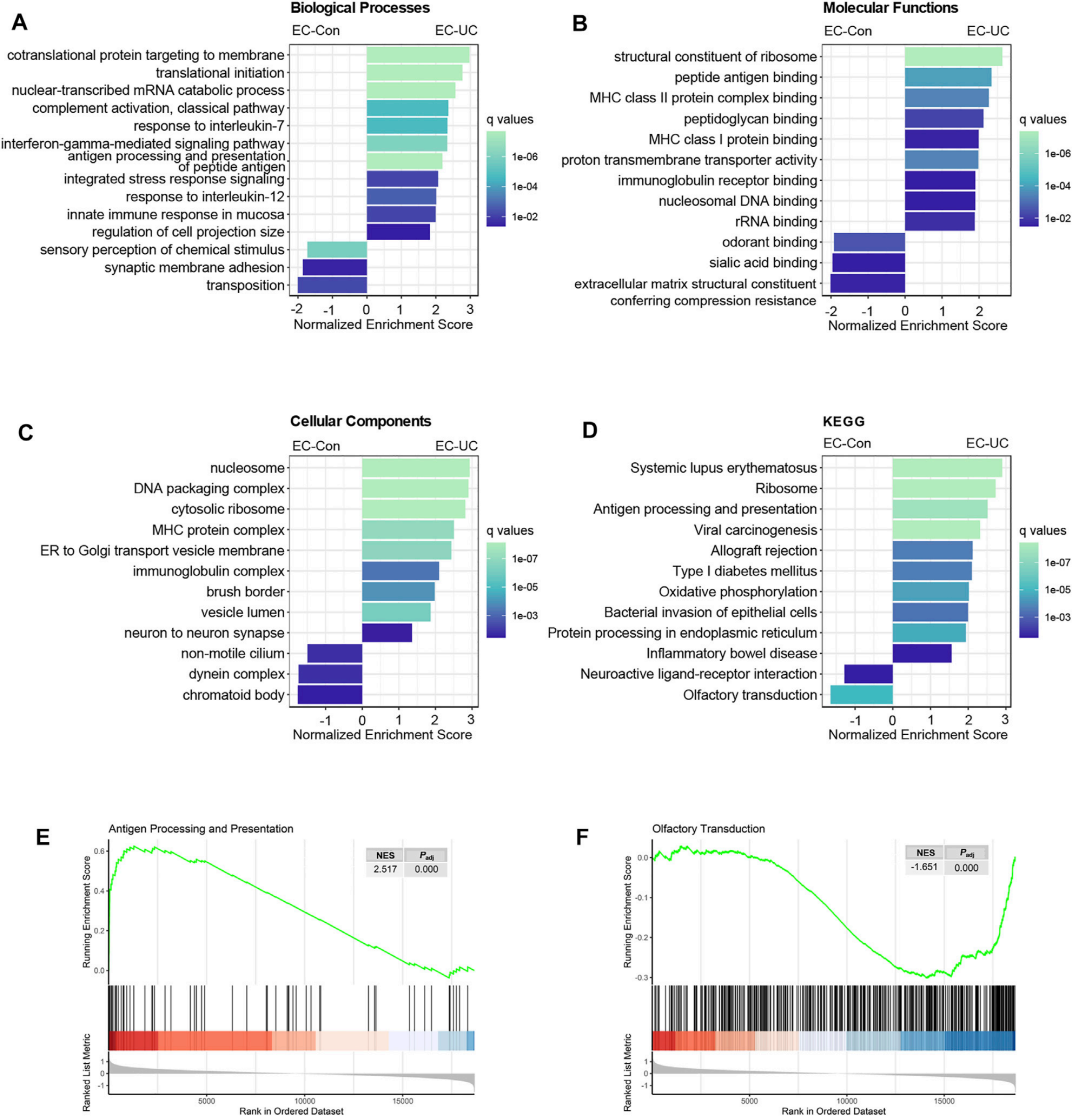
Enrichment analysis revealing a functional shift of EC cells in patients with UC. **(A–D)** Results of the GSEA comparing EC-UC and EC-Con. Analysis was performed using the Gene Ontology categories of biological process **(A)**, molecular function **(B)**, and cellular component **(C)**, and KEGG pathways **(D)**. The length of each bar indicates the normalized enrichment score, and the color indicates q values of each term. **(E,F)** GSEA enrichment plot of two KEGG pathways: “antigen processing and presentation” and “olfactory transduction.” NES, normalized enrichment score. *P*_adj_, adjusted *p*-value.

To identify functional changes in EC cells under UC conditions, we conducted GSEA using both GO terms and KEGG pathways. Genes annotated with GO terms associated with protein synthesis, such as “cotranslational protein targeting to membrane,” “translation initiation,” and “nuclear-transcribed mRNA catabolic process,” were enriched in EC cells from patients with UC (Figures 5A–C), indicating upregulation of protein synthesis in UC. Genes annotated with GO terms related to humoral immunity, such as “complement activation,” “antigen processing and presentation,” and “innate immune response in mucosa,” were also enriched in this group, and the MHC I and MHC II pathways were both activated (Figures 3A,B,E). Terms associated with the immune system molecules IL-7, IL-12, and IFN-γ were also enriched. Interestingly, genes annotated with the GO terms “regulation of cell projection size” and “neuron to neuron synapse” were upregulated as well (Figures 3A,C), probably associated with changes in the neuropods extending from EC cells.

KEGG pathway analysis revealed enrichment of pathways associated with autoimmune diseases other than inflammatory bowel disease, such as systemic lupus erythematosus, allograft rejection, and type I diabetes mellitus (Figure 5D); this suggested an intrinsic connection between UC and other autoimmune diseases.

Both GO term analysis and KEGG pathway analysis showed that the most prominently downregulated pathways were those associated with chemical and olfactory sensation (Figures 5A,B,D,F). Humans possess more than 1,000 kinds of olfactory receptors, some of which are expressed in EC cells as well as in olfactory tissues. We found downregulation of 140 genes in the olfactory-related terms in EC cells from patients with UC, although none of them were statistically significant (Supplementary Table S3).

### Data Validation Through Single-Cell RNA Sequencing and Immunofluorescence Staining

**FIGURE 6:**
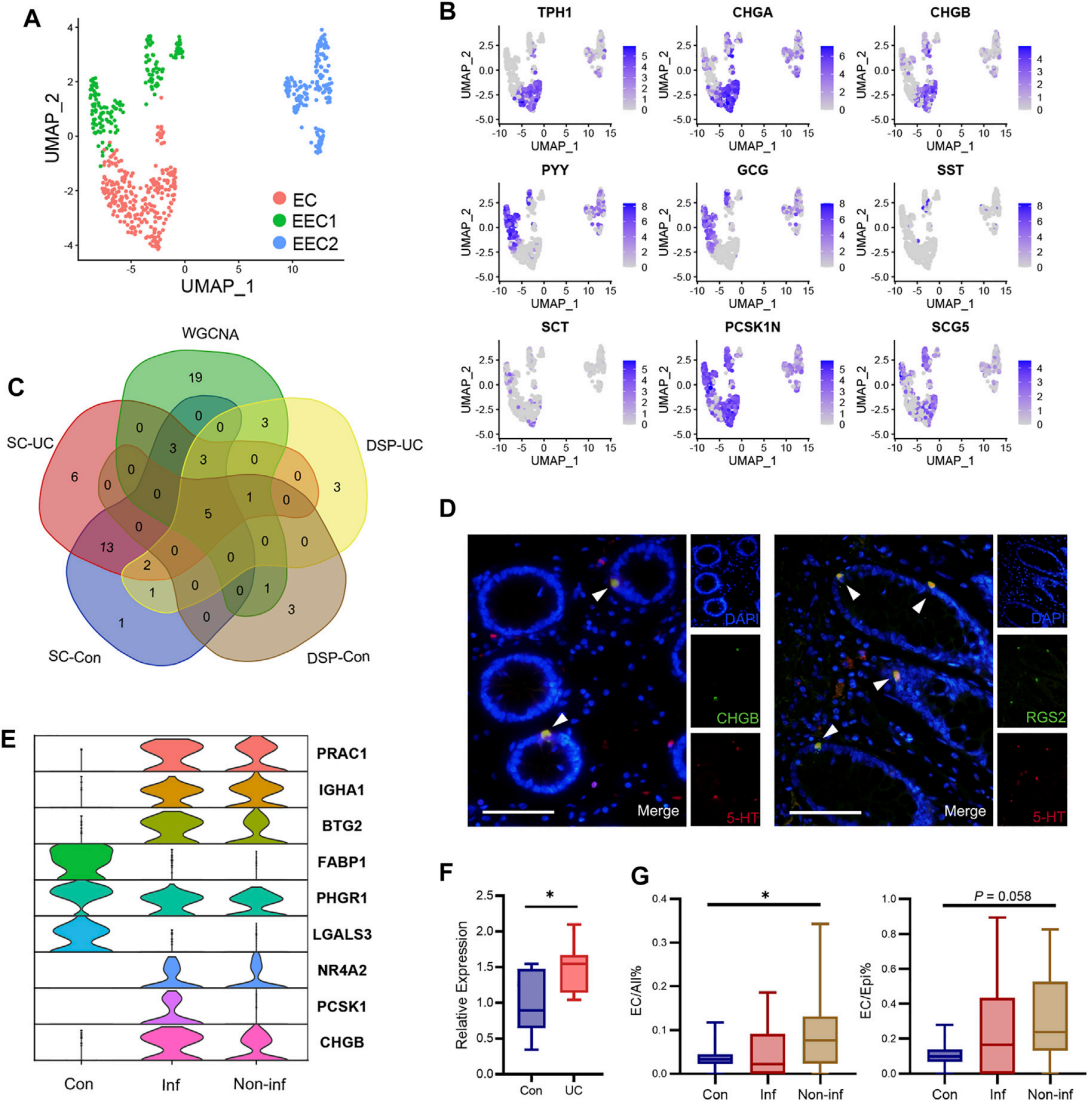
Data validation using single-cell transcriptomic data and immunofluorescence staining. **(A)** UMAP (Uniform Manifold Approximation and Projection) plot of enteroendocrine cells, including 271 EC cells, 167 cells for the EEC1 group, and 166 cells for the EEC2 group. **(B)** Markers of enteroendocrine cells plotted on the UMAP plot. **(C)** Venn diagram of genes enriched in EC cells as determined by single-cell RNA-seq, genes enriched in EC cells as determined by DSP, and genes in the Green3 module associated with EC cells as identified by WGCNA. **(D)** Representative immunofluorescence staining of CHGB and RGS2 in colonoscopic biopsy samples from healthy participants. The results indicated a significant co-localization of EC cells (5-HT positive), and CHGB and RGS2, respectively. Scale bar = 100 μm; magnification, ×400. **(E)** Violin plot of DEGs between EC cells from the inflamed mucosa of patients with UC and controls. Con, control. Inf, inflamed mucosa of patients with UC. Non-inf, non-inflamed mucosa of patients with UC. **(F)** The expression level of CHGB in patients with UC was confirmed by qRT-PCR. Differences between groups were analyzed using Student’s *t*-test (two-tailed). *p < 0.05. **(G)** Boxplot showing the ratios of EC cells to all cells (left) and to epithelial cells (right).

To validate our findings, we imported an online dataset containing single-cell transcriptomic data of colonic cells from 18 patients with UC and 12 healthy controls (Smillie et al., 2019). After QC, we were left with a dataset of 118,233 epithelial cells clustered into 26 subsets (Supplementary Figure S2A). Cluster 22 was identified as consisting of enteroendocrine cells based on their expression of *TPH1*, *CHGA*, and *PCSK1N* (Supplementary Figure S2B). To identify EC cells, the enteroendocrine cells were isolated and clustered into three subsets (Figure 6A). The enteroendocrine cells expressed various enteroendocrine markers, as described by a previous study (Gehart et al., 2019), with each subset showing a distinctive spectrum of secretion (Figure 6B). Cells were identified as EC cells if they were high in *TPH1*, low in *PYY*, and low in *GCG*.

Differential expression analysis of genes enriched in EC cells found 28 genes enriched in healthy controls, 33 in inflamed UC mucosa, and 43 in non-inflamed UC mucosa (Supplementary Table S4). Our UC samples for DSP were from inflamed mucosa; therefore, we compared the overlap between the genes enriched in EC cells as determined by single-cell RNA-seq, those enriched in EC cells as determined by DSP, and those in the coexpression module associated with EC cells as identified by WGCNA (Figure 6C). Nine genes specifically enriched in EC cells (*RGS2*, *PCSK1N*, *CHGB*, *CHGA*, *TPH1*, *CRYBA2*, *SCG5*, *IGFBP3*, and *DDC*) were found in at least four subsets above. *RGS2*, *PCSK1N*, *CHGB*, *CHGA*, and *TPH1* were found in every subset. Apart from *TPH1*, *RGS2* also showed high specificity to EC cells (Supplementary Figure S2C). The expression of *RGS2* and *CHGB* in EC cells was further validated by immunofluorescence staining (Figure 6D). There were also 26 genes in the coexpression network of EC cells that were not included in the genes in Figure 6C. We then investigated whether their expression was specific to EC cells, using both our DSP data and the single-cell RNA-seq data. Although not statistically significant, those genes showed an enrichment tendency in EC cells in DSP data (Supplementary Figure S2D). In the single-cell RNA-seq data, the expression of *SLC38A11* and *C6orf141* was specific to EC cells, whereas *GUCY2C*, *INSM1*, *MS4A8*, *GCLC*, *PTPRN2*, *CPE*, *RAB3B*, *UCP2*, *NEU4*, *QDPR*, and *SCGN2* were specifically expressed in enteroendocrine cells (Supplementary Figure S2E).

Because of the low number of EC cells, differential expression analysis comparing inflamed UC mucosa and control found only nine genes that were significantly differentially expressed (Figure 6E, Supplementary Table S4), among which *CHGB* was also upregulated in our DSP data. We further confirmed the upregulation of CHGB in patients with UC by qRT-PCR (Figure 6F). The top eight upregulated genes in UC as determined by DSP were detected in very few of the EC cells analyzed by single-cell RNA-seq (Supplementary Figure S2F). *ITLN1*, *LCN2*, and *OLFM4* were expressed in cluster EEC2 and some other epithelial cells, whereas *REG1A* was only detected in cluster 16 (Supplementary Figure S2G). *IGHG1*, *IGHG2*, *IGHG3*, and *IGHG4* were not detected in the single-cell RNA-seq data. This was probably due to a difference in biopsy location.

We also performed a between-group comparison of the ratios of EC cells to background epithelial cells and to all cells (Figure 6G). Compared to healthy controls, non-inflamed mucosa from patients with UC showed an increased proportion of EC cells.

## Discussion

In this study, we profiled the transcriptomic patterns of EC cells in both patients with UC and healthy individuals using DSP. Our findings indicate that EC cells possess greater capacity for protein synthesis and hormone secretion than other cell clusters in the epithelium. These results provide the first evidence that, in UC, EC cells increase protein synthesis and hormone secretion while reducing their role in the sensation of chemical stimuli. Our findings increase the knowledge of the biological behavior of EC cells under both healthy and UC conditions.

Previous studies have demonstrated that EC cells have multiple functions apart from 5-HT secretion. Single-cell profiling has revealed that EC cells produce various hormones, including GIP, ghrelin, PYY, GLP-1, secretin, and somatostatin (Reynaud et al., 2016; Martins et al., 2017; Fazio Coles et al., 2020). Moreover, various receptors for chemical (Kidd et al., 2008; Akiba et al., 2015) and mechanical (Alcaino et al., 2018) stimuli have been identified in EC cells, and there is evidence of direct communication between EC cells and adjacent neuronal synapses (Bellono et al., 2017). In our study, EC cells showed a distinctive expression pattern associated with the synthesis and secretion of protein, which may enable them to produce sufficient peptide hormones to fulfill their function as enteroendocrine cells. In addition to genes involved in 5-HT production, we also found that genes encoding various secretory hormones or peptides, including *CHGA*, *CHGB*, and *SCG5*, were expressed in EC cells. The proteins CHGA, CHGB, and SCG5 are common neuroendocrine markers mainly found in transport vesicles (Bartolomucci et al., 2011). To our knowledge, this is the first study to report enrichment of *RGS2* and *SLC38A11* in EC cells; however, its role remains unknown. EC cells have multiple functions and play a vital role in the homeostasis of the gastrointestinal tract; however, many of their functions remain to be elucidated.

There is increasing evidence that the behavior of EC cells is substantially altered in UC, particularly with regard to elevated levels of 5-HT and CHGA (El-Salhy et al., 1997; Sciola et al., 2009). Single-cell RNA-seq has been applied to investigate UC in several studies (Parikh et al., 2019; Smillie et al., 2019; Li et al., 2021). A recent study (Parikh et al., 2019) compared enteroendocrine cells in patients with UC with healthy controls, finding that the former showed enrichment in five genes (*HMGCS2*, *FABP1*, *CD74*, *HLA-C*, *SERPINH1*, and *B2M*) and in antigen presentation pathways. Three of these genes (*CD74*, *HLA-C*, and *B2M*) were also enriched in our EC-UC group. Notably, our GSEA results reveal a previously unobserved functional shift, in which EC cells under UC conditions produce more proteins and hormones and gain the capacity for antigen processing and presentation while downregulating their role in sensing chemical signals *via* olfactory receptors. Pathway analysis also indicated changes in EC cells’ neuropods and synaptic communication. It is widely accepted that EC cells play an important part in mucosal immunity *via* the secretion of 5-HT (Wang F. et al., 2017; Eissa et al., 2018; Ito et al., 2019). This study provides evidence that EC cells directly take part in antigen presentation *via* both MHC I and MHC II pathways, which is consistent with a previous study (Parikh et al., 2019). The secretion of 5-HT by EC cells is reportedly regulated *via* olfactory receptors and neuronal communication. Changes in these functions provide a possible reason for the overproduction of 5-HT and other enteroendocrine hormones in patients with UC.

As a member of the chromogranin family, CHGB is the precursor of several secretory peptides; its function in the gastrointestinal system remains largely unknown. Previous studies have found increased fecal CHGB in patients with UC in remission (Strid et al., 2013), and patients with UC show higher serum levels of CHGB than patients with Crohn’s disease (Wasinger et al., 2016). However, the tissue origin of increased CHGB levels in patients with UC remains unknown. In this study, we found significantly increased levels of CHGB in the EC cells of patients with UC, confirmed by both DSP, single-cell RNA-seq data and qRT-PCR. However, these results were inconsistent with those obtained by conducting the same comparison using some bulk RNA-seq datasets from Gene Expression Omnibus (data not shown). This could be due to inconsistencies in the number of EC cells in the UC mucosa, which could be significantly influenced by the inflammation level of the biopsy location. Further study on the role of CHGB and its derived peptides in UC, especially at the protein level, is still needed.

Our study only examined samples from the large intestine and was therefore unable to illuminate the function of EC cells in other parts of the gastrointestinal tract. Moreover, further functional assays are still needed to confirm the functional change of EC cells in UC. In addition, some genes identified in prior studies as being specifically expressed in EC cells, such as *Piezo2* and *TGR5*, did not appear in our list of DEGs, probably due to limited sample size or differences in biopsy location.

Despite the limitations of our study, the results clearly indicate the distinctive expression patterns of EC cells in UC. EC cells undergo a functional shift from sensation to secretion and antigen presentation, along with an upregulation of *CHGB*. Thus, this study provides a deeper understanding of the behavior of EC cells, as well as suggesting novel targets for UC treatment.

## Data Availability

The datasets presented in this study can be found in online repositories. The names of the repository/repositories and accession number(s) can be found below: https://www.ncbi.nlm.nih.gov/geo/, GSE183853.
