# Supplementary material for: Digital Spatial Profiling Reveals Functional Shift of Enterochromaffin Cell in Patients With Ulcerative Colitis
Source: Front Cell Dev Biol. 2022 Apr 8;10:841090. doi: 10.3389/fcell.2022.841090 (PMC9023741; doi:10.3389/fcell.2022.841090)
Supplement: Supplementary file 5 [file DataSheet1.docx]

Supplementary Material
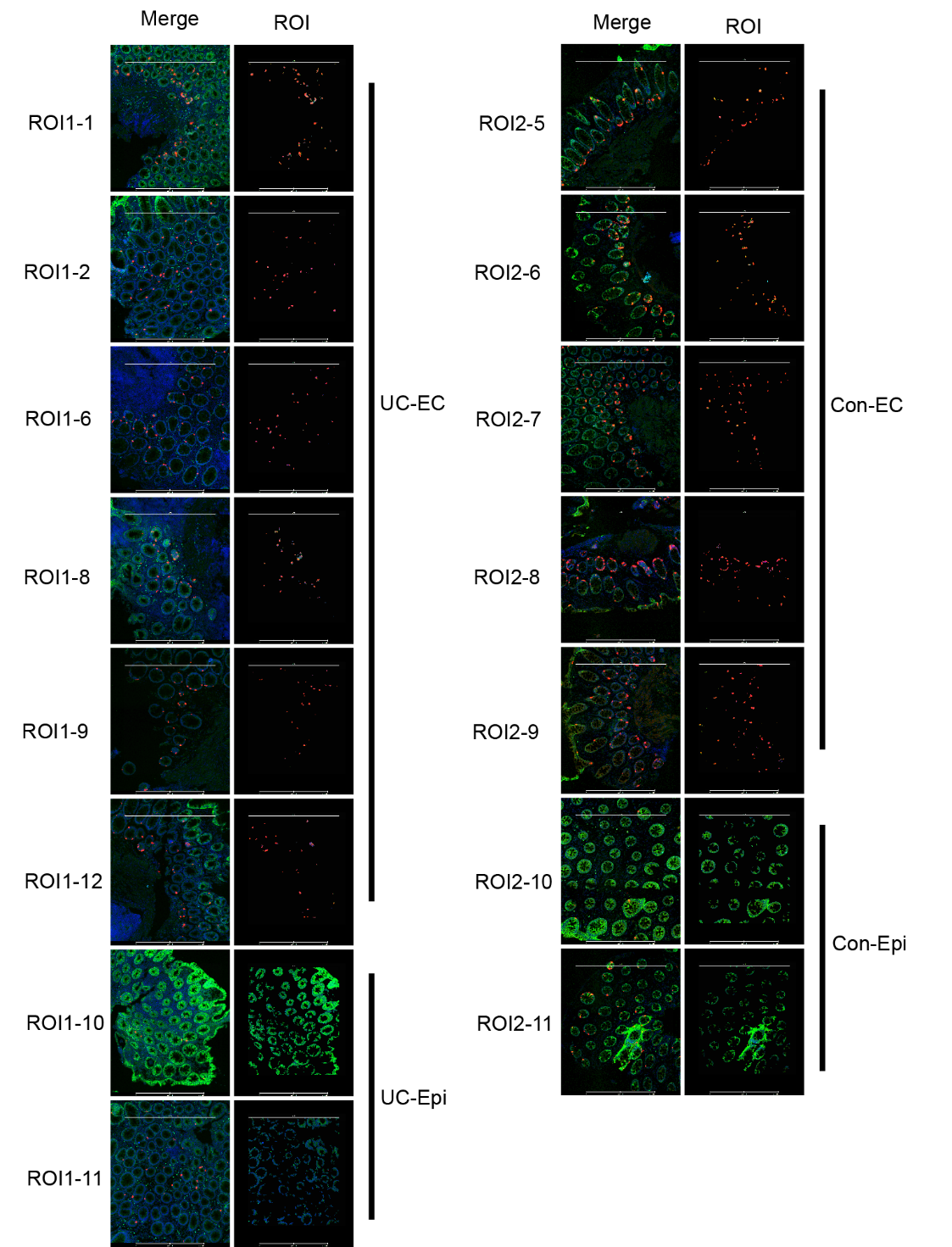


**Supplementary Figure 1.** Immunofluorescent image of each ROI. ROIs were selected based on the immunofluorescence signals. The EPCAM+ 5-HT+ areas were for EC cells, while EPCAM+ 5-HT− areas for background epithelium. Red, anti-5-HT. Green, anti-EpCAM. Blue, Syto13.

**
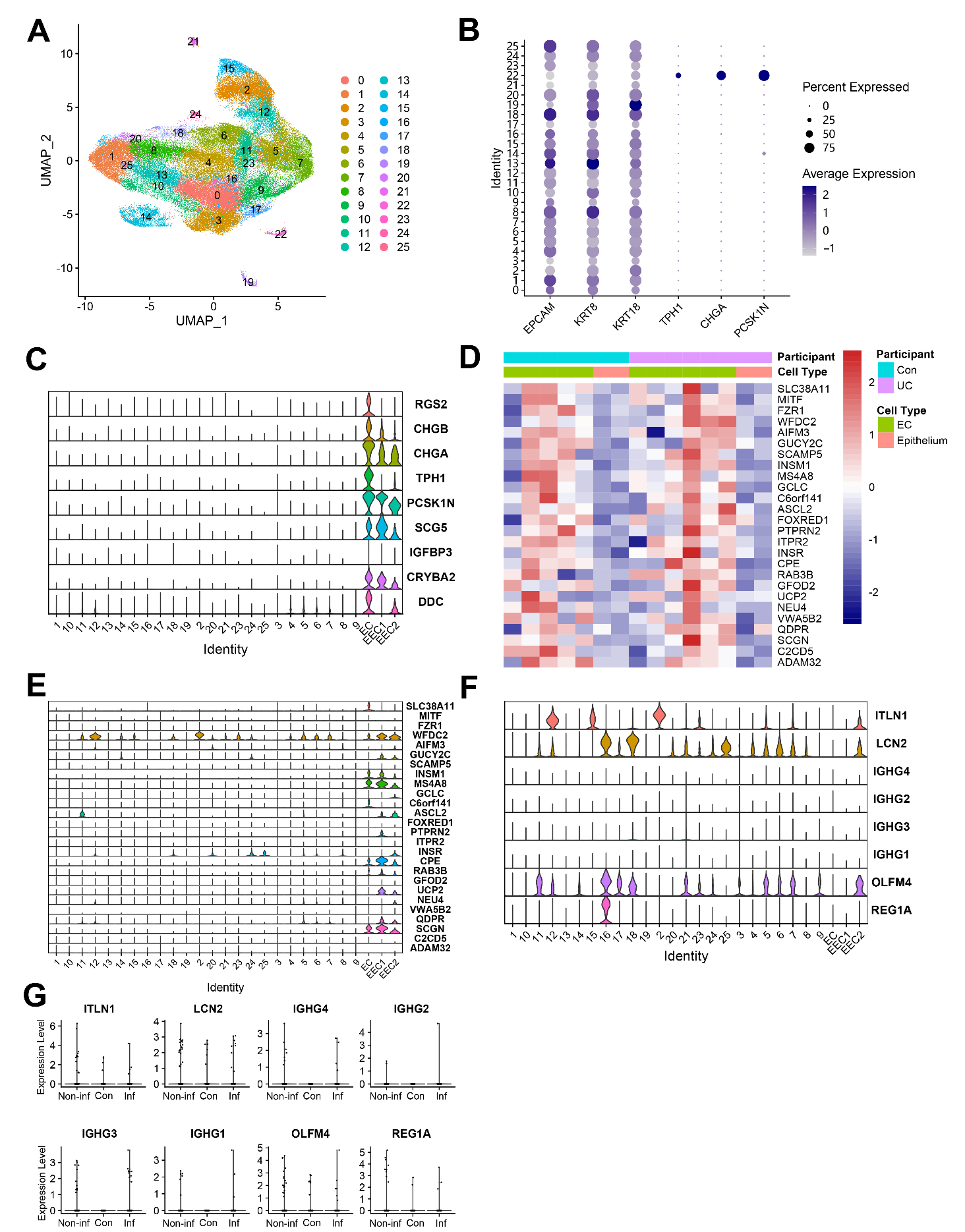
**

**Supplementary Figure 2.** Data validation using single-cell transcriptomic data.

(A) UMAP plot of epithelial cells after integration and clustering. A total of 118,233 epithelial cells were clustered into 26 subsets.

(B) Dot plot showing the expression of enteroendocrine cell markers (*TPH1*, *CHGA*, *PCSK1N*) and epithelial markers (*EPCAM*, *KRT8*, *KRT18*; positive controls) in each cluster. Size of each dot indicates the percentage of cells expressing the corresponding target, and the color indicates the average expression level of the target.

(C) The expression of genes included in at least four subsets in Figure 6C in each cluster of the single cell RNA-seq data.

(D,E) The expression of genes in the co-expression network of EC cells but not included in the EC-related genes in (C), shown in our DSP data (D) and in each cluster of the single cell RNA-seq data (E).

(F) The top eight up-regulated genes in the EC-UC group as determined by our DSP data were expressed in very few cells in the single-cell RNA-seq data. Con, control. Inf, inflamed mucosa of UC patients. Non-inf, non-inflamed mucosa of UC patients.

(G) The expression level of genes in (F) in EC cells from control, inflamed or non-inflamed mucosa of UC.
